# The prevalence of psychiatric comorbidities in epileptic patients

**DOI:** 10.1192/j.eurpsy.2023.1594

**Published:** 2023-07-19

**Authors:** H. Berrada, H. Chebli, K. Hajjami, S. Belbachir, A. Ouanass

**Affiliations:** Hôpital psychiatrique arrazi de Salé, Sale, Morocco

## Abstract

**Introduction:**

Habituellement, nous voyons dans la consultation psychiatrique des patients épileptiques pour des plaintes psychologiques. Souvent pour un trouble anxieux ou dépressif. Dans d’autres cas, nous prenons en charge des patients psychiatriques atteints d’épilepsie.

**Objectives:**

L’objectif de notre travail est d’étudier la prévalence de la comorbidité psychiatrique dans une population de patients épileptiques.

**Methods:**

Nous avons mené une étude transversale auprès de la consultation de l’hopital ar-razi de salé, à l’aide d’un questionnaire et d’échelles d’évaluation préétablies : le MINI (mini entretien neuropsychiatrique international), l’inventaire de la dépression de Beck, et Échelle d’anxiété de Hamilton.

**Results:**

55 patients ont répondu aux critères d’inclusion. L’âge moyen de nos patients est de 25,9 ans. Le sex-ratio F/H est de 1,6. Plus de la moitié de nos patients sont célibataires (78%). Seuls 21 % des patients ont une activité professionnelle régulière. La prévalence des troubles psychiatriques dans notre étude est de 66,6%. L’anxiété est retrouvée dans 68,2 % des cas, alors que la dépression est évaluée à 58,9 % des cas dont 23,7 % ont une dépression sévère qu’il faut traiter, avec une prédominance du sexe féminin (66,8 %).

**Conclusions:**

Dans notre étude, l’ anxiété et la dépression sont les troubles psychiatriques les plus rapportés en cas d’épilepsie. Ils doivent être systématiquement recherchés pour tout épileptique et traités pour améliorer la qualité de vie des patients.

**Disclosure of Interest:**

None Declared

